# Supplementary material for: Performance and Stability of Tenofovir Alafenamide Formulations within Subcutaneous Biodegradable Implants for HIV Pre-Exposure Prophylaxis (PrEP)
Source: Pharmaceutics. 2020 Nov 5;12(11):1057. doi: 10.3390/pharmaceutics12111057 (PMC7694512; doi:10.3390/pharmaceutics12111057)
Supplement: Supplementary file 1 [file pharmaceutics-12-01057-s001.pdf]

pharmaceutics

MDPI

*Supplementary Materials*

# Performance and Stability of Tenofovir Alafenamide Formulations within Subcutaneous Biodegradable Implants for HIV Pre-Exposure Prophylaxis (PrEP)

**Linying Li, Leah M. Johnson, Sai Archana Krovi, Zach R. Demkovich and Ariane van der Straten**

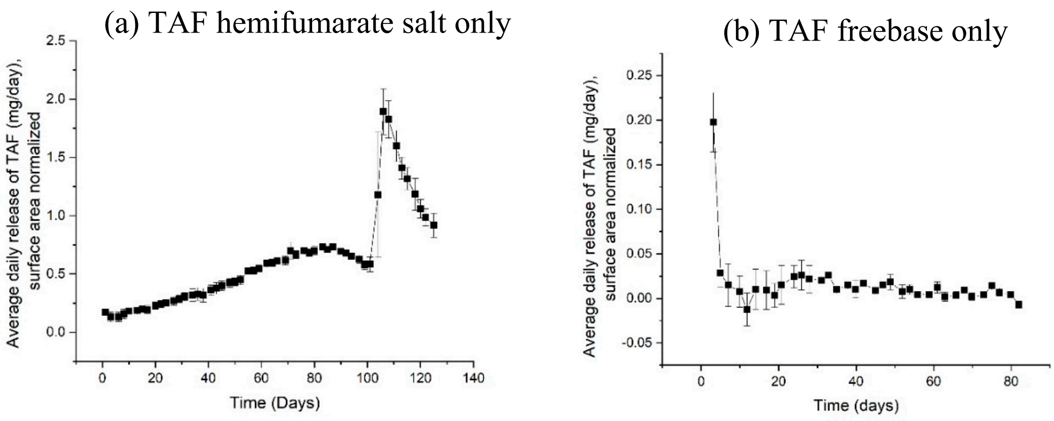


**Figure S1.** Daily release profiles of implants filled with TAF hemifumarate salt ($TAF_{HF}$) (**a**) and TAF freebase ($TAF_{FB}$) (**b**).

**Table S1.** Chromatographic purity of $TAF_{HF}$ formulated with castor oil or PEG600 inside implants exposed to simulated physiological conditions over 120 days.

| Chromatographic purity | $TAF_{HF}$-Castor oil | $TAF_{HF}$-PEG600 |
|---|---|---|
| **Time 0** | 99.8% | 99.7% |
| **Day 14** | 99.3% | 97.3% |
| **Day 28** | 98.9% | 94.8% |
| **Day 42** | 97.4% | 89.7% |
| **Day 52** | 96.0% | 76.0% |
| **Day 62** | 94.7% | 63.3% |
| **Day 82** | 82.6% | 40.3% |
| **Day 92** | 74.4% | 22.9% |
| **Day 102** | 57.1% | 10.3% |
| **Day 120** | 17.6% | 1.36% |

**Table S2.** Chromatographic purity of $TAF_{FB}$ formulated with sesame oil or castor oil inside implants exposed to simulated physiological conditions over 210 days.

| | **$TAF_{FB}$-Sesame oil** | **$TAF_{FB}$-Castor oil** |
|---|---|---|
| **Day 0** | 99.8% | 99.8% |
| **Day 90** | 98.4% | 97.1% |
| **Day 120** | 98.0% | 96.5% |
| **Day 150** | 96.0% | 92.5% |
| **Day 180** | 94.9% | 88.5% |
| **Day 210** | 92.0% | 84.5% |

**Table S3.** Water ingress within the implant reservoir of $TAF_{HF}$ formulated with castor oil or PEG600 measured via the loss-on-drying method.

| **Formulations** | **$TAF_{HF}$-Castor oil** | | | **$TAF_{HF}$-PEG600** | | |
|---|---|---|---|---|---|---|
| **Timepoint** | **Water content (mg)** | **% water ingress (of total mass of implant)** | **Cumulative drug release (mg)** | **Water content (mg)** | **% water ingress** | **Cumulative drug release (mg)** |
| **Time 0** | 0 | 0% | 0 | 2.0 | 2% | 0 |
| **Day 14** | 0.6 | 0.7% | 4.2 | 6.7 | 8.1% | 10.2 |
| **Day 28** | 1.0 | 1.2% | 8.3 | 7.8 | 9.4% | 16.1 |
| **Day 42** | 1.5 | 1.8% | 15.4 | 8.4 | 10.1% | 26.4 |
| **Day 52** | 2.1 | 2.4% | 19.6 | 8.6 | 10.3% | 38.0 |
| **Day 62** | 3.0 | 3.6% | 22.1 | 13.1* | 15.8% | 54.5 |
| **Day 82** | 4.0 | 4.8% | 30.6 | 10.9 | 13.1% | 52.6 |
| **Day 92** | 5.5 | 6.6% | 35.6 | 13.2* | 15.9% | 74.8 |
| **Day 102** | 8.2* | 9.8% | 50.5 | 13.3* | 16.0% | 56.7 |
| **Day 120** | 9.7 | 11.6% | 44.6 | 18.0* | 21.6% | 102.9 |

* Implants are compromised.

**Table S4.** Water ingress within the implant reservoir of $TAF_{FB}$ formulated with castor oil or sesame oil measured via loss-on-drying method.

| Formulations | $TAF_{FB}$-Sesame oil | | | $TAF_{FB}$-Castor oil | | |
|---|---|---|---|---|---|---|
| **Timepoint** | **Water content (mg)** | **% water ingress (of total mass of implant)** | **Cumulative drug release (mg)** | **Water content (mg)** | **% water ingress** | **Cumulative drug release (mg)** |
| **Time 0** | 0 | 0% | 0 | 0% | 0 | 0 |
| **Day 90** | 5.0 | 2.1% | 16.9 | 11.7 | 5.1% | 22.0 |
| **Day 120** | 10.0 | 4.6% | 21.6 | 19.6 | 8.4% | 29.8 |
| **Day 150** | 15.1 | 6.9% | 26.7 | 29.1 | 12.2% | 38.3 |
| **Day 180** | 22.7 | 10.6% | 32.0 | 50.1 | 20.5% | 47.6 |
| **Day 210** | 35.3 | 15.4% | 38.2 | 55.4 | 23.1% | 60.1 |

**Table 5.** Chromatographic purity profile of $TAF_{HF}$ castor oil and $TAF_{FB}$ sesame oil implants stored in open and closed foil pouches stored at 22 °C/50% RH and 40 °C/75% RH over 6-months.

| Formulation | Storage Condition | Day 0 | Day 90 | Day 180 |
|---|---|---|---|---|
| **$TAF_{HF}$-Castor oil** | 22°C/50% RH *(sealed)* | 99.5% | 99.3% | 99.1% |
| | 22°C/50% RH *(open)* | 99.5% | 99.3% | 99.0% |
| | 40°C/75% RH *(sealed)* | 99.5% | 98.8% | 97.5% |
| | 40°C/75% RH *(open)* | 99.5% | 97.3% | 91.2% |
| **$TAF_{FB}$-Sesame oil** | 22°C/50% RH *(sealed)* | 99.6% | 99.1% | 98.0% |
| | 22°C/50% RH *(open)* | 99.6% | 98.5% | 98.6% |
| | 40°C/75% RH *(sealed)* | 99.6% | 98.8% | 98.0% |
| | 40°C/75% RH *(open)* | 99.6% | 99.4% | 97.5% |
